# Comparative effectiveness of different platelet-rich plasma for arthroscopic rotator cuff repair: A protocol for systematic review and network meta-analysis

**DOI:** 10.1097/MD.0000000000031260

**Published:** 2022-10-21

**Authors:** Weiwei Shen, Wei Wang, Yun Xue, Jie Shi, Zhongshu Pu, Qiuming Gao

**Affiliations:** a Orthopedics Center of PLA, The 940th Hospital of Joint Logistics Support Force Army of PLA, Lanzhou, China; b Department of Orthopedics, The Third People’s Hospital, Baiyin, China; c Department of Infection Control, The 940th Hospital of Joint Logistics Support Force Army of PLA, Lanzhou, China.

**Keywords:** arthroscopic rotator cuff repair, network meta-analysis, platelet-rich plasma, protocol, randomized controlled trials

## Abstract

Rotator cuff tears are one of the most common injuries of the shoulder joint in adults. Arthroscopic rotator cuff repair (ARCR) has become the gold standard for the treatment of rotator cuff injuries, but it still has a high rate of retear. Platelet-rich plasma (PRP) has been widely used as an adjunct to ARCR. However, the comparative efficacy of different PRP for ARCR remain unclear. In order to evaluate the relative efficacy of PRP for individuals with ARCR, we will conduct a systematic review and network meta-analysis of randomized controlled trials.

**Methods:**

A systematic literature search will be conducted in Embase, PubMed, Cochrane Library, Web of Science, China National Knowledge Infrastructure, Wanfang Database, and Chinese Biomedical Literature Database will be searched up to October 2022. The primary outcome will focus on the retear rate at the last follow-up. The secondary outcomes include the Visual Analogue Scale for postoperative pain and functional capacity scores. The risk of bias for individual studies will be assessed according to the revised Cochrane risk-of-bias tool for randomized trials (RoB 2.0). Data analysis will be performed using *R* 4.1.2. Publication bias will be examined using comparison-adjusted funnel plots and Egger’s test using STATA 15.0. The quality of evidence will be assessed using the Grading of Recommendations, Assessment, Development and Evaluation approach.

**Results:**

The results of this study will be submitted to a peer-reviewed journal for publication.

**Conclusions:**

The review will compare the efficacy of different PRP for patients with ARCR. The result of the study will provide evidence-based medical evidence for ARCR with PRP augmentation.

## 1. Introduction

Rotator cuff tears are one of the most common injuries of the shoulder joint in adults, which seriously reduces the quality of work and life of patients.^[1]^ Arthroscopic rotator cuff repair (ARCR) can effectively relieve pain and improve the function scores of patients. It has become the gold standard for the treatment of rotator cuff injuries,^[2]^ but the postoperative retear rate varies between 5% and 51%.^[3]^ The retear substantially affects patients’ satisfaction and dramatically increases the probability of a second operation. It has been demonstrated that the scar tissue formed at the tendon-bone interface is far inferior to the normal tissue.^[4]^ Therefore, in recent years, the focus of current research has shifted from mechanical repair alone to mechanical repair in conjunction with biological enhancement to improve the postoperative healing rate of ARCR.^[5]^

As an autologous platelet concentrate, platelet-rich plasma (PRP) can release a large number of bioactive factors, effectively improving tissue regeneration and healing ability.^[6]^ PRP has been widely used in the arthroscopic repair of rotator cuff tears, but its clinical effect is still unclear, and even conflicting results.^[5]^ The absence of a clear consensus on the efficacy of PRP is likely attributable to the different types of PRP used in the various studies.^[5,7]^ Dohan Ehrenfest et al classified PRP into four categories: pure PRP (P-PRP), leukocyte-rich PRP (L-PRP), pure platelet-rich fibrin (P-PRF), and leukocyte-rich platelet fibrin (L-PRF).^[8]^ Unfortunately, no systematic comparison so far has been performed to identify the most comparatively effective PRP for patients with ARCR. Unlike conventional pairwise meta-analyses, network meta-analysis (NMA) combine direct and indirect evidence to evaluate the relative efficacy of multiple interventions.^[9]^ Therefore, in this study we will conduct an NMA to evaluate the efficacy of various PRP augmentation for ARCR. We believe this work will provide evidence-based medical evidence for the treatment of rotator cuff tears with PRP augmentation and offer better assistance for clinical practice.

## 2. Methods

### 2.1. Study design and registration

We registered the research on PROSPERO (registration number: CRD42022351759) and will conduct our systematic review according to the Preferred Reporting Items for Systematic Reviews and Meta-Analysis Protocol (PRISMA-P) guidelines.^[10]^ The PRISMA-P checklist is shown in the online supplemental materials S1, http://links.lww.com/MD/H686. Furthermore, the review will be reported in adherence with the PRISMA extension statement for incorporating network meta-analysis (PRISMA-NMA).^[11]^

### 2.2. Inclusion criteria

#### 2.2.1. Type of studies.

Only randomized controlled trials (RCTs) published in English or Chinese will be included. The following types of papers will be excluded: qualitative studies, editorials, reviews, opinion papers and case studies. Non-experimental studies such as cohort and case–control studies will also be excluded.

#### 2.2.2. Types of participants.

Our systematic review will include all patients diagnosed with rotator cuff tears by MRI or ultrasound examination, and administered with ARCR, regardless of age, sex, and tear size. However, if significant subgroup differences are discovered between rotator cuff tear sizes, we will conduct a subgroup analysis to further explore this heterogeneity.

#### 2.2.3. Types of interventions and comparisons.

Eligible interventions must have one specific kind of PRP augmentation, including P-PRP, L-PR, P-PRF, and L-PRF. There will be no restriction on PRP types (gel vs liquid), PRP activating agents (calcium gluconate vs calcium chloride), time of PRP application (intraoperative vs postoperative), PRP dose, and times of PRP administration. The patients who received ARCR treatment alone will be included as a contrast group in this systematic review. The treatment arm will be considered regardless of surgical repair types (single-row or double-row).

#### 2.2.4. Types of outcome measure.

As primary outcomes, the postoperative retear rate will be analyzed within a follow-up period of at least six months. The most common modality to evaluate tendon healing was magnetic resonance imaging. Retear was defined as absence of visible tendonfiber extending across the entire repaired tendon as type IV and V according to the Sugaya classification.^[12]^ The secondary outcomes will include the Visual Analogue Scale for postoperative pain and functional capacity scores. The latter contains the Constant Score,^[13]^ the shoulder rating scale of the University of California at Los Angeles (UCLA) score,^[7]^ simple shoulder test (SST) score.^[14]^

### 2.3. Data sources and search strategy

RCTs will be searched systematically up to October 2022 in the following databases: Embase, PubMed, Cochrane Library, Web of Science, China National Knowledge Infrastructure, Wanfang Database, and Chinese Biomedical Literature Database. The publication languages will be limited to English or Chinese. Searches will combine the free text words and MeSH terms regarding “Platelet-Rich Plasma” and “Rotator Cuff Injuries” to identify target trials. The search strategy for PubMed is provided in the online supplemental material S2, http://links.lww.com/MD/H687. Corresponding search strategies will be modified for other databases as required. The reference lists from eligible studies and relevant systematic reviews will be searched manually.

### 2.4. Study selection

**Figure 1.**
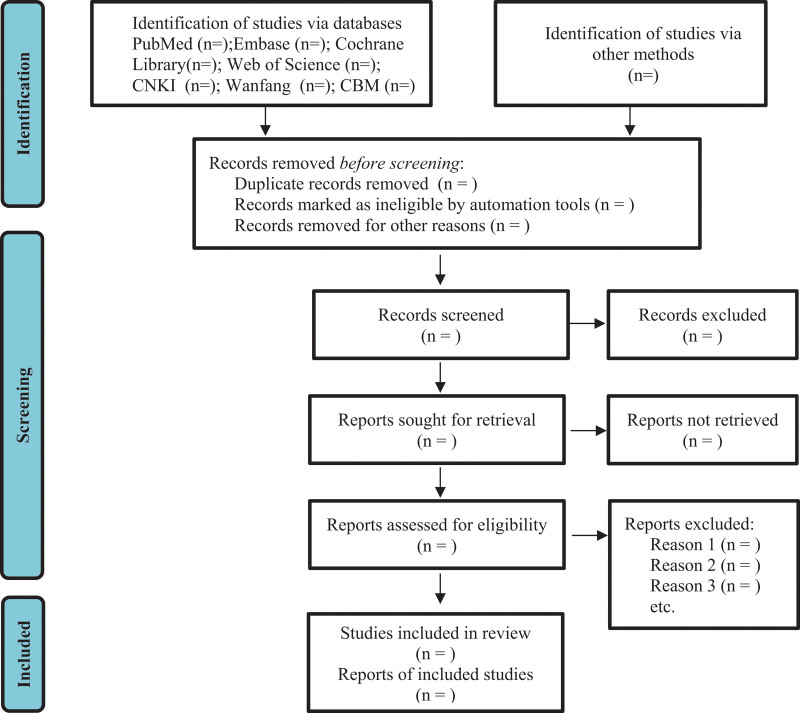
Flow diagram of study selection process.

All retrieved studies will be imported into Endnote X9, and duplicates will be removed. Titles and abstracts will be screened through an initial search by two reviewers independently. After excluding irrelevant publications, another two reviewers will download the full text of all potentially relevant studies for further independent assessment. We will review the full text of the remaining publications against the same eligibility criteria. Any disagreement will be resolved through team discussion or consultation with a third reviewer. Finally, the numbers of studies identified, excluded (with the reason for exclusion) and included in the systematic review and subsequent meta-analysis will be summarized using the Preferred Reporting Items for Systematic Reviews and Meta-Analyses flow diagram (Fig. 1).^[15]^

### 2.5. Data extraction

Two authors will independently extract data from all include studies using a standard data abstraction sheet. Any disputes will be resolved by discussion until the consensus is reached or by consulting a third investigator.

The following data will be extracted:

1. General information: title, authors, country, the language of publication, the year of publication, sponsors.
2. Trial characteristics: study design, total study duration, sequence generation, allocation sequence concealment, and blinding method.
3. Participants: diagnostic criteria, total number, age, gender, country, rotator cuff tear sizes.
4. Interventions: PRP type, activating agent, application time, PRP dose, and times of PRP administration.
5. Outcomes: all specified primary and secondary outcomes, follow-up time, number of participants with complete follow-up and reasons for loss to follow-up.

Data estimates (e.g., mean, SD) that may be accessed visually from figures of publications will be extracted using Engauge Digitizer 10.8 software (Mitchell 2016). If both SD and SE are missing but *P* values or CIs are available, we will calculate SD according to the Cochrane Handbook guidelines.

### 2.6. Risk of bias assessment

Based on the revised Cochrane risk-of-bias tool for randomized trials (RoB 2.0), the methodological quality of each included study will be assessed independently by two reviewers.^[16]^ This tool consists of five domains including the randomization process, deviations from intended interventions, missing outcome data, measurement of the outcome and selection of the reported result. All Studies will be evaluated according to the following response options for the signaling questions: “no” or “probably no” (considered as high risk), “yes” or “probably yes” (considered as low risk) and no information (indicated some concerns). Finally, each study will be given an overall grade of high risk, moderate risk or low risk of bias. A third reviewer will resolve any disagreement through discussion if necessary.

### 2.7. Statistical analysis

#### 2.7.1. Pairwise meta-analysis.

We will perform the pairwise meta-analysis on direct comparisons with *R* 4.1.2 software using the meta package.^[17]^ The mean difference or standardized mean difference with 95% confidence intervals (CIs) will be calculated for continuous data. Odds ratio (OR) with 95% CI will be calculated for dichotomous data. The statistical heterogeneity across studies will be assessed using the *I*^2^ statistics.^[18]^ *I*^2^ values over 50% will indicate considerable heterogeneity, and then a random-effects model will be used. Otherwise, a fixed-effected model will be applied.

#### 2.7.2. Network meta-analysis.

We will perform network meta-analysis on direct and indirect comparisons with *R* 4.1.2 software using GeMTC and ggplot2 package.^[19]^ Random-effects modes will be adopted in this network meta-analysis, as they are considered to be the most conservative approach to dealing with between-study heterogeneity. Mean difference or standardized mean difference and 95% CI will be calculated for continuous variables, while OR with 95% CI will be calculated for dichotomous outcomes. We will use Markov chain Monte Carlo simulations with 50,000 iterations in which the first 20,000 iterations will be abandoned as burn-in. The model convergence will be examined with the Gelman-Rubin-Brooks diagnostic plots and potential scale reduction factor (PSRF).^[20]^ Afterward, in the case of closed loops of interventions, the node-splitting method will be used to estimate the inconsistency by comparing the direct evidence with the indirect evidence.^[21]^ Meanwhile, the surface under the cumulative ranking curve will be calculated to obtain the ranking probability of the different interventions.^[22]^ We report surface under the cumulative ranking curve as percentages, where a score closer to 100% represents a greater chance of that treatment being the best among all treatments studied for that outcome.

### 2.8. Subgroup and sensitivity analyses

If heterogeneity among the studies is detected, subgroup analysis will be performed according to the rotator cuff tear sizes, PRP type, PRP activating agent, surgical repair type, and other relevant parameters. We will also conduct sensitivity analyses by removing each study 1 at a time to evaluate the stability of the results. If sensitivity analysis shows a fundamental change in the heterogeneity or the findings of meta-analysis, then the stability of the meta-analysis will be determined as poor.

### 2.9. Evidence quality assessment

We will rate the quality of evidence of the network meta-analysis results according to the Grading of Recommendations, Assessment, Development and Evaluation (GRADE) group with GRADE Pro software.^[23]^ The certainty of the evidence for each important outcome will be evaluated as very low, low, moderate, or high. The detailed information and certainty of the evidence will be reported in the “Summary of findings” tables.^[24]^

### 2.10. Assessment of publication biases

Publication bias will be examined by inspection of comparison-adjusted funnel plots based on all included trials using STATA 15.0.^[25]^ Funnel plots provide a visual aid for detecting publication bias in systematic reviews. Furthermore, Egger’s test will be performed to formally confirm the existence of publication bias.^[26]^ If the funnel plots are found to be asymmetrical, and the publication bias is identified, we will attempt to explain the asymmetry.

## 3. Discussion

Rotator cuff tears are common causes of pain and shoulder joint disability.^[1]^ ARCR has been recognized as a major treatment approach, as it can effectively relieve pain and improve function, with less surgical trauma. However, a higher postoperative retear rate after ARCR substantially affects patient function and satisfaction.^[3]^ Platelet-rich plasma (PRP) has been applied as an adjunct to rotator cuff repair to improve tendon-bone healing and potentially reduce the incidence of subsequent tendon retear.^[27]^ However, there are many controversies regarding the application of autologous PRP, potentially because of the different types of PRP used in the various studies.^[28,29]^

Currently, several conventional pairwise meta-analyses have investigated the comparative efficacy of single PRP intervention for ARCR.^[30–32]^ However, the comparative efficacy of different PRP interventions in patients with ARCR is not yet clear. No NMA has been performed to evaluate the comparative efficacy of all the available PRP interventions. To the best of our knowledge, this review is the first to use Bayesian mesh meta-analysis on the basis of existing RCTs and to evaluate and rank the most effective PRP interventions for ARCR. Our results will strengthen the understanding of the benefit of each individual PRP, and will provide evidence-based medical evidence of clinical rational PRP interventions for the treatment of rotator cuff tear.

However, this study also has some limitations. It is difficult to rule out heterogeneity completely because of the differences in rotator cuff tear sizes, surgical repair types, PRP intervention dose and frequency, follow-up time, etc. Therefore, more high-quality, multicenter RCTs are needed to further confirm the effectiveness of PRP interventions for ARCR.

## Author contributions

**Conceptualization:** Weiwei Shen, Qiuming Gao.

**Data curation:** Wei Wang, Yun Xue.

**Methodology:** Weiwei Shen, Zhongshu Pu.

**Project administration:** Qiuming Gao.

**Software:** Weiwei Shen, Jie Shi.

**Writing – original draft:** Weiwei Shen, Yun Xue.

**Writing – review & editing:** Weiwei Shen, Qiuming Gao.

## Supplementary Material


