# Supplementary material for: Comparative effectiveness of different platelet-rich plasma for arthroscopic rotator cuff repair: A protocol for systematic review and network meta-analysis
Source: Medicine (Baltimore). 2022 Oct 21;101(42):e31260. doi: 10.1097/MD.0000000000031260 (PMC9592368; doi:10.1097/MD.0000000000031260)
Supplement: Supplementary file 2 [file medi-101-e31260-s002.pdf]

The search strategy for PubMed

#ID	Topic or intervention	Query
#1	Disease	"Platelet-Rich Plasma"[Mesh] OR Platelet Rich Plasma[Title/Abstract] OR PRP[Title/Abstract] OR Platelet-Rich Plasma[Title/Abstract] OR thrombocyte rich plasma[Title/Abstract] OR platelet-rich plasma cell[Title/Abstract] OR platelet-rich fibrin [Title/Abstract] OR PRF[Title/Abstract]
#2	Intervention	Rotator Cuff Injury[Title/Abstract]) OR (Rotator Cuff Tears[Title/Abstract] OR (Rotator Cuff Tear[Title/Abstract])) OR (Rotator Cuff Injuries[MeSH Terms] OR (Rotator Cuff Tendinosis[Title/Abstract])) OR (Supraspinatus [Title/Abstract]
#3	Study design	"randomized controlled trial"[pt] OR "controlled clinical trial"[pt] OR randomized[tiab] OR placebo[tiab] OR "drug therapy"[sh] OR randomly[tiab] OR trial[tiab] OR groups[tiab])
#4	Final query	#1 AND #2 AND #3
